# 

**Published:** 1869-02

**Authors:** 


### Books and Pamphlets Received.

Treatise on the Diseases of the Ear, including the Anatomy of the Organ. By Anton Von Tröltsch, M. D., Professor in the University of Würzburg, Bavaria. Translated and edited by D. B. St. John Roosa, M. A., M. D., Clinical Professor of the Diseases of the Eye and Ear in the University of New York, Surgeon to the Brooklyn Eye and Ear Hospital, formerly Surgeon to the New York Eye and Ear Infirmary, etc. Second American from the fourth German edition. New York: Wm. Wood & Co., 1869. Received through Breed & Lent, Buffalo.

Pennsylvania Hospital Reports. Vol. II, 1869. Philadelphia: Lindsay & Blakiston. Received through Theo. Butler & Son, Buffalo.

A Hand-Book of Uterine Therapeutics, and of Diseases of Women. By Edward John Tilt, M. D., member of the Royal College of Physicians; Consulting Physician to the Faringdon General Dispensary; Fellow of the Royal Medical and Chirurgical Society, and of several British and foreign societies. Second American edition, thoroughly revised and amended. New York: D. Appleton & Co., 1869. Received through Breed & Lent, Buffalo.

A History of the Medical Department of the University of Pennsylvania from its foundation in 1765, with sketches of the lives of deceased Professors. By Joseph Carson, M. D., Professor of Materia Medica and Pharmacy in the University of Pennsylvania; member of the American Philosophical Society, etc. Philadelphia: Lindsay & Blakiston, 1869. Received through Theo. Butler & Son, Buffalo.

Transactions of the Medical Society of the State of New York, for the year 1868. Albany: Van Benthuysen & Son, 1868.

Library of Education; selected from the best writers of all countries; some thoughts concerning Education. By John Locke. First edition. New York: J. W. Schermerhorn & Co., 1869.

On the Microscope in the Diagnosis and Treatment of Sterility. By J. Marion Sims, M. D. Reprint from the New York Medical Journal of January, 1869. New York: D. Appleton & Co., 1869.

Archives of Ophthalmology and Otology. Edited and published simultaneously in English and German, by Prof. H. Knapp, M. D., in New York, and Prof. S. Moos, M. D., in Heidelberg. New York: Wm. Wood & Co., 1869.

Report of the Board of Directors of the Eastern Lunatic Asylum for the fiscal year ending September 30th, 1868.

Joined Twins; the Obstetrical and Surgical Management, with remarks. By A. B. Cook, A. M., M. D., Professor of the Principles and Practice of Surgery in the Kentucky School of Medicine, Louisville. Louisville: Bell & Co., 1869.

A new Operation for Artificial Hip-joint in Bony Anchylosis. Illustrated by two cases. Reprint from Transactions of the Medical Society of the State of New York for 1863, with additional notes and letters from eminent medical gentlemen. By Lewis A. Sayre, M. D., Surgeon to the Bellevue Hospital, Professor of Orthopedic Surgery, Bellevue Hospital Medical College. New York: D. Appleton & Co., 1869.

Accidental and Congenital Atresia of the Vagina, with a Mode of Operation for successfully establishing the canal. By Thomas Addis Emmet, M. D., Surgeon in charge of the New York State Women's Hospital. New York: Bradstreet press, 1866.

First Annual Report of the New York Orthopedic Dispensary, located at 1299 Broadway, New York, 1869.

Rush Medical College. Annual Announcement of the Spring Term for 1869. The session commences Wednesday, March 3d.

